# Health monitoring at the Robert Koch Institute – Effects of a change in study design on sample composition and prevalence estimates due to the start of the ‘Health in Germany’ Panel

**DOI:** 10.25646/13567

**Published:** 2025-12-05

**Authors:** Elvira Mauz, Felicitas Vogelgesang, Stefan Damerow, Anja Schienkiewitz, Niels Michalski, Beate Gaertner, Jennifer Allen, Jens Baumert, Yong Du, Ronny Kuhnert, Johannes Lemcke, Ramona Scheufele, Angelika Schaffrath Rosario

**Affiliations:** Robert Koch Institute, Department of Epidemiology and Health Monitoring, Berlin, Germany

**Keywords:** Survey design, Surveillance, Change in methodology, Prevalence comparison, Response behaviour, Representativeness, Bias, Correction

## Abstract

**Background:**

The Robert Koch Institute (RKI) continuously monitors key health indicators in the general population by collecting data repeatedly. As changes in survey design can affect prevalence estimates and thus make interpreting trends difficult, the launch of the RKI Panel ‘Health in Germany’ was accompanied by a methodological study.

**Methods:**

The RKI Panel is based on a random sample drawn from population registers. The survey is self-administered in written format (online or paper). The composition of the sample, prevalence estimates and response behaviour were then compared with data collected in parallel in the GEDA 2024 telephone survey. Data from previous surveys were included in the modelling to quantify method-related differences in prevalence estimates.

**Results:**

The RKI Panel 2024 was more successful in representing young adults, the elderly and individuals with low levels of education. The prevalence estimates differ significantly from GEDA 2024 in some cases, particularly for mental health indicators and their associated factors. The RKI Panel includes more older adults with limited physical health, while in young adults more participants with poorer mental health are present. Despite method-related differences in prevalence, modelling can usually be used to assess trends.

**Conclusions:**

The RKI Panel provides a more realistic representation of the German population than previous telephone surveys. The differences in prevalence are due to effects of the survey mode, questionnaire design, and changes in sample composition.

## 1. Introduction

As part of its health monitoring activities, the Robert Koch Institute (RKI) continuously collects key indicators that describe the health status of the general population [1]. This includes both one-off surveys on health topics that have not yet been sufficiently researched and the systematic development of time series for selected indicators, which enable an assessment of their development in different population groups. Since 2024, regularly updated results have been published on the web portal of Federal Health Reporting and gradually supplemented with additional indicators [2].

To provide reliable health reporting data on an ongoing basis and in crisis situations, a new panel infrastructure was introduced at the RKI in 2024 [3]. The RKI Panel ‘Health in Germany’ addresses key challenges in epidemiological surveys, such as declining participation rates, increasing costs and difficulty of reaching certain population groups, particularly the elderly and people with low levels of education [4–9]. The aim is to ensure the regular collection of questionnaire, examination and laboratory data using consistent methodology in the long term, and to enable to implement necessary surveys quickly and flexibly in crisis situations [3]. Before the establishment of the RKI Panel, various health surveys had been conducted since 1984 [1], which varied over time in terms of their sampling design and survey mode. While some surveys were based on random samples from residents’ registration offices in nationwide survey locations (registry samples), others used telephone samples from landline numbers, and later from both landline and mobile numbers (dual-frame samples). Data collection was carried out partly by telephone and partly in writing (online or paper) (Table 1). The RKI Panel is based on an registry sample and a written mixed-mode design (online or paper survey) [3], like the GEDA 2014/2015-EHIS study [10].

To interpret temporal trends correctly, it is important to use a survey methodology that is as constant as possible, since methodological changes can affect the comparability of prevalence estimates and lead to divergent results [11–13]. These method-related effects can influence both the willingness to participate, thereby affecting the representativeness of the sample (selection effects), and the response behaviour, thereby affecting the estimation of proportions (measurement effects; see overview in [14, 15]). The change in study design from the GEDA studies (‘German Health Update’) of recent years to the RKI Panel involves the following changes: (1) changing from a dual-frame telephone sample to a registry sample, (2) changing in the survey mode from telephone interviews to a self-administered written mixed-mode survey, (3) updating the weighting procedure, and (4) dividing the questionnaire thematically into four modules. Accordingly, effects on the sample structure, response behaviour and consequently on the prevalence estimates were assumed.

To continue the time series started on the health reporting web portal and to enable reliable interpretation despite the change in methodology, methodological studies were carried out to accompany the launch of the RKI Panel: (1) A telephone study (GEDA 2024) was conducted in parallel with the first annual survey of the RKI Panel (RKI Panel 2024), in the summer of 2024. This enabled a comparison of sample composition and prevalence estimates of identically surveyed indicators between the ‘old’ and ‘new’ study designs. (2) Additionally, data from previous surveys were used to identify, describe, and quantify the effects of the design change on the prevalence of health indicators over time, where possible.

**Key messages**

► The effects of the change from the GEDA study to the RKI Panel were assessed in a methodological study.
► The change in methodology can significantly alter prevalence estimates, particularly for mental health indicators.
► The RKI Panel provides a better representation of Germany’s population structure than the previous GEDA studies.
► Population groups such as young adults, the elderly aged 80 and older, and those with low levels of education are better represented in the RKI Panel.
► Topics with a tendency towards socially desirable responses, subjective assessments and gradual response options (Likert scales) are particularly susceptible to method effects.

The paper aims to assess and quantify the method effects of the change in survey design, ensuring reliable interpretation of the time series of health indicators. The development of correction factors is intended to make prevalence estimates from different survey designs comparable. An indicator sheet documenting the effects of the new survey methodology and showing the correspondingly adjusted prevalence is provided for each indicator presented on the web portal of Federal Health Reporting.

## 2. Methods

The RKI studies used for the methodological analyses were divided into two study types based on the main differences in sampling and survey mode (Table 1):

1. Studies with a registry sample and a written mixed-mode design were assigned to study type 1. This includes the newly established RKI Panel.
2. Studies based on a telephone sample (landline or dual-frame sample) and telephone interviews were assigned to study type 2.

### 2.1 Data sources

#### Panel ‘Health in Germany’ – recruitment study and RKI Panel 2024 (study type 1)

The RKI Panel ‘Health in Germany’ was established in 2024 through a recruitment study. The population for recruitment was persons aged 16 and older who were living in the Federal Republic of Germany during the survey period, regardless of their nationality or country of birth. However, only German-language survey instruments were used. Sampling was based on a two-stage stratified random selection. First, 359 primary sampling units (sample points), were randomly selected from all municipalities in Germany, considering the regional structure. In the second stage, addresses were drawn from the address registers of the respective residents’ registries for each sample point, stratified by age group. Approximately 167,000 individuals were invited to participate in a short recruitment survey and asked to consent to participating in future surveys as part of the RKI Panel [3]. The recruitment rate was 29 %. The survey was conducted among individuals under the age of 70 using a sequential mixed-mode design, in which a link and a QR code to the online survey were included in the invitation letter. If they do not participate, a paper questionnaire was offered in addition to online participation in the second reminder letter. For people aged 70 and over, a simultaneous mixed-mode design was used, whereby participants could choose between an online or paper questionnaire when they received the invitation to participate in the study. This approach was also continued in up to two reminder letters [16].

In the first annual survey in 2024, the RKI Panel comprised 46,863 registered participants aged 18 and over: 24,881 women, 21,856 men, and 126 individuals identifying as non-binary. They were invited to complete health surveys at three time points (quarter 2 – Q2, quarter 3 – Q3 and quarter 4 – Q4) at intervals of approximately two months. A total of four different questionnaires (A, B, C and D) were used, each covering different topics, meaning the topics were less mixed than in previous RKI surveys. Panel members received one of the four questionnaires in each of the three sub-waves, in a predefined rotating procedure, so that each person received three of the four questionnaires. Additionally, the first subwave questionnaires contained in-depth socio-demographic questions at the end. Participants could respond online or on paper; the mode used in the recruitment survey determined the mode for the 2024 annual wave. Data collection began in May 2024 with the first sub-wave and was completed in early January 2025 with the third sub-wave. The re-participation rate (the proportion of participants in relation to the number of people registered in the panel) in the individual sub-waves was between 75 % and 81 %, in accordance with the standards of the American Association for Public Opinion Research (AAPOR [17]). A detailed description of the methodology and response rates (also stratified by age and gender) can be found elsewhere [18].

#### Data basis for the methodological study: Parallel surveys GEDA 2024 (study type 2) and summer months (Q2 + Q3) of the RKI Panel 2024 (study type 1)

The first two sub-waves of the RKI Panel 2024 (Q2 and Q3) were accompanied by the GEDA 2024 methodological study, which used the same design as previous GEDA telephone surveys. GEDA 2024 was based on a telephone sample of people aged 18 and over and was conducted via telephone interviews using a dual-frame approach. Compared to previous GEDA waves, a significantly reduced survey inventory was used, with the selected content collected in the same way as for the RKI Panel 2024 (see 2.2). The survey was conducted between June and September 2024, with approximately 1,000 participants per month (n = 4,016; response rate 20 %). For the comparative analyses, data from the first two of the RKI Panel 2024’s three sub-waves (quarters 2 and 3, n ≈ 18,000 depending on the respective questionnaire) were used.

#### Trend analyses: Previous RKI Health Monitoring Surveys

To assess the effects of the methods over time and to calculate correction factors using statistical modelling, additional RKI surveys were included and assigned to the two types of study being compared:

Study type 1: GEDA 2014/2015-EHIS was assigned to this study type with a sample drawn from population registers, and a sequential written mixed-mode design that had been comprehensively tested in advance [19, 20]. Initially, only an online questionnaire was offered, and a paper questionnaire was only added as a reminder. This design is similar to that of the RKI Panel; the only difference is that the RKI Panel offered a simultaneous mixed-mode design directly to people aged 70 and over.

Study type 2: All other health monitoring survey studies correspond to study type 2 with telephone samples and telephone interviews. Surveys conducted up to 2012 (GSTel 2003 and 2006; GEDA 2009, 2010 and 2012) were based exclusively on landline samples. From 2019/2020 onwards, dual-frame samples, i.e. landline and mobile phone, were used (2019/2020, 2022, and 2023).

#### Weighting in the RKI surveys

A weighting factor was constructed for each RKI survey, for the analysis. This takes the respective sample design into account and compensates for selective participation as far as possible.

Weighting of the RKI Panel: To correct for bias due to selective participation and deviations of the sample from the population structure, a multi-stage sample weight was calculated. This is based on the sample weight of the initial recruitment study. In addition, drop-out weights were calculated using the recruitment study data to compensate for selective participation in the repeated sub-waves. Finally, the sample was adjusted to official population figures as of 31 December 2023 and to the 2021 Microcensus. Age, gender, federal state, BIK municipality type [21], education groups (according to the Comparative Analysis of Social Mobility in Industrial Nations, CASMIN [22]) and household size (single vs. multi-person household) were taken into account. Information on education was collected as part of the recruitment study. Information on household size was collected via the sociodemographic questionnaire in the first sub-wave of the annual survey. If this information was missing (e.g. due to non-participation in the first sub-wave), it was replaced by the corresponding information from the recruitment study. The weighting was calculated separately for each questionnaire type. A detailed methodological description is provided in a separate article [23].

Weighting of previous surveys: All previous RKI surveys have already been weighted for earlier analyses (see Annex Table 1 for details). Similarly, the parallel GEDA 2024 survey was weighted by age, gender, federal state, district type, and education (based on the population as of 31 December 2023 and according to the 2021 Microcensus). The previous weighting differed from the weighting in the RKI Panel in two ways: (1) weighting by household size was introduced for the first time in the RKI Panel and (2) the distribution of education in the RKI Panel was adjusted according to the CASMIN classification [22] following [24], whereas in previous RKI surveys it was adjusted according to the ISCED classification (International Standard Classification of Education [25]).

### 2.2 Health indicators

A total of eleven indicators were examined for method effects. These indicators were collected as self-reported data in both the RKI Panel 2024 survey and earlier RKI surveys, thus enabling statements about trends (Table 1). Additional data from the parallel GEDA 2024 survey is available for seven of these indicators. The indicators were included in two different questionnaires in the RKI Panel 2024: Questionnaire A focused on indicators of physical health and risk factors, while Questionnaire C focused on indicators of mental health and its influencing factors, as well as fatigue and exhaustion.

To formulate hypotheses about the expected direction of prevalence differences between interview-based and self-administered surveys, the indicators were classified according to factors known from the literature to influence response behaviour (e.g. [19, 26–28]), namely (1) degree of social desirability, (2) question type (factual vs. subjective) and (3) scale level of the response format (Annex Table 2).

#### Indicators of physical health and risk factors

##### General state of health

The Minimum European Health Module (MEHM) [29, 30], an integral part of the RKI health monitoring system, was used to record three indicators:

The indicator *Self-rated general health* was defined as the proportion of adults who rated their general state of health as ‘very good’ or ‘good’. This was recorded with the question ‘How is your health in general?’ (possible answers: very good | good | fair | bad | very bad).

The indicator *Chronic conditions* was recorded using the following question: ‘Do you have any chronic disease or a long-term health problem? This means disease or health problems that have lasted or are expected to last for at least 6 months.’ (possible answers: Yes | No).

The indicator *Long-term activity limitations* was defined as the proportion of adults who have been severely limited or limited in performing usual activities due to a health problem for at least six months. The question was: ‘Are you limited because of a health problem in activities people usually do?’ (Possible answers: Severely limited | Limited, but not severely | Not limited at all). Those who were limited were also asked how long the limitations had lasted (possible answers: Less than 6 months | 6 months or longer).

##### Impairments in physical function

The *Impairments in physical function* indicator reflects severe mobility impairment, and was defined as the proportion of adults who have a lot of difficulties walking or climbing stairs, or are unable to do so. It was assessed using two questions from the European Health Interview Survey [31]: ‘Do you have difficulty walking half a kilometre (500 metres) on level ground without the use of any aid?’ and ‘Do you have difficulty walking up or down 12 steps?’ (Possible answers: No difficulty | Some difficulty | A lot of difficulty | Cannot do at all/Unable to do). A mobility impairment exists if at least one of the two forms of mobility is performed with a lot of difficulty or cannot be performed at all.

##### Diabetes mellitus

The indicator *Diabetes mellitus* was defined as the proportion of adults who have ever had a physician-diagnosis of diabetes. Women who only reported a diagnosis of gestational diabetes were excluded. Participants in the various surveys were asked, with almost identical wording, whether they had ever been diagnosed with diabetes by a doctor (possible answers: yes | no).

##### Obesity

*Obesity* was defined as a body mass index (BMI) ≥ 30 kg/m^2^ [32]. BMI was calculated from participants’ self-reported body weight and height. The wording of the question varied slightly across the different surveys, with responses recorded in centimetres for height and in kilograms for weight [33].

##### Smoking

The *smoking* indicator was based on the question about current tobacco product smoking status, with slightly different wording across the surveys, but always with the same response options [33]: Yes, daily | Yes, occasionally | No, no longer | Have never smoked. Typically, the prevalence of current smoking (Yes, daily | Yes, occasionally) is shown. In the RKI Panel 2024, this question was only asked to people without diabetes mellitus. For people with diabetes, information from the recruitment study was used instead.

#### Indicators of mental health and its determinants

##### Self-rated mental health

The indicator *self-rated mental health* was defined as the proportion of adults who rated their mental health as generally excellent or very good [34]. An internationally established single item was used: ‘How would you describe your mental health in general?’ (possible answers: excellent | very good | good | not so good | poor).

##### Depressive symptoms

*Depressive symptoms* in the population were assessed using the Patient Health Questionnaire-8 (PHQ-8) [35]. Participants were asked how frequently they had felt bothered by eight symptoms of major depression in the previous two weeks. The possible answers to the eight items were assigned values: not at all (value 0) | on some days (value 1) | on more than half of the days (value 2) | almost every day (value 3). These values are added up to give a total score ranging from 0 to 24. A screening score of 10 or greater indicates potentially clinically relevant symptoms of depression.

##### Anxiety symptoms

The indicator for *anxiety symptoms* in the population was measured using the Generalised Anxiety Disorder-2 (GAD-2) scale [36]. Participants were asked how frequently they had felt bothered by two core symptoms of generalised anxiety disorder over the previous two weeks. The possible answers and corresponding values are analogous to those of the PHQ-8 and add up to a total score between 0 to 6. A screening score of 3 or more indicates potentially clinically relevant symptoms of anxiety.

##### Social support

*Social support* was measured using the Oslo 3-item Social Support Scale (OSSS-3), which measures the perceived availability of social support [37]. Specifically, the questions asked were: ‘How many people are so close to you that you can count on them if you have serious problems?’ (possible answers: None | 1 to 2 | 3 to 5 | 6 or more), ‘How much concern and interest do people show in what you are doing?’ (possible answers: A lot of concern and interest | Some concern and interest | Uncertain | Little concern and interest | No concern and interest) and ‘How easy is it to get practical help from neighbours in case of need?’ (possible answers: Very easy | Easy | Possible | Difficult | Very difficult). Adding the individual scores from the three questions creates an index that can range from 3 to 14 points. A score of 12 to 14 points is classified as strong support.

### 2.3 Statistical analysis of parallel surveys in 2024

The two surveys conducted simultaneously, GEDA 2024 and RKI Panel 2024 (limited to quarters 2 and 3), were compared in terms of (1) sample composition; (2) prevalence of health indicators; and (3) response behaviour. Only individuals aged 18 and over were included in the analysis. The analyses employed the weighting factors and, for the RKI Panel, took clustering in sample points into account using SAS 9.4 survey procedures of (SAS Institute, Cary, NC, USA). For the RKI Panel 2024, the weighting factors created for the entire annual survey (not restricted to Q2 and Q3) were used. Both surveys were weighted to the population as of 31 December 2023. For stratified analyses by education level, standardisation was carried out within the education groups using the European Standard Population [38], ensuring that these analyses were based on the same age and gender distribution for all education levels.

#### Sample composition

To assess the sample composition in both surveys, the following were examined: weighted and unweighted proportions as well as the proportion of missing values for gender (based on current gender identity), five age groups (18 – 29, 30 – 44, 45 – 64, 65 – 79, 80 +), three education groups (according to the CASMIN classification: low, medium and high [22]) and household size. To test whether differences in the sample composition could be explained by changes in weighting procedures for the RKI Panel, a sensitivity analysis was conducted on the GEDA 2024 data, which was weighted in the same way as the RKI Panel (i.e. taking household size into account and using the CASMIN education classification instead of the ISCED education classification).

#### Comparing the prevalence of health indicators

To compare prevalence estimates, weighted proportions are presented for the total population and for subgroups stratified by gender, age and education groups (according to the CASMIN classification). However, the comparison does not distinguish between effects due to changes in sample composition, survey mode or questionnaire design. The effects of the change in weighting were examined in a sensitivity analysis, in which GEDA 2024 was weighted in the same way as the RKI Panel.

#### Response behaviour depending on the survey mode and response format

The indicators differ depending on their question and answer format. These formats are influenced to varying degrees by the change in survey mode [26, 27, 39]. The indicators were therefore classified according to the format of the individual items on which they are based (categorical, single or multiple Likert scale, or metric response) and compared with each other. In addition, the proportions of missing values were compared depending on the response format.

### 2.4 Analysis of temporal trends in prevalence and estimation of the method effects

The differences observed in the direct prevalence comparison between the parallel GEDA 2024 and RKI Panel 2024 surveys are subject to greater statistical uncertainty, particularly in subgroups, due to the smaller number of cases in GEDA 2024. Therefore, prevalence estimates from previous surveys were included in the analysis (Table 1, Annex Table 1) to estimate the magnitude of the method-related effect and better assess the trend.

However, when estimating the method effect, it was not possible to distinguish between effects due to changes in sample composition, survey mode, questionnaire design or weighting. Instead, the overall method effect was estimated by comparing the prevalence trends between the two study types (i.e. study type 1 vs. study type 2). A model was established for each indicator using logistic regression, including the survey date (average participation date per survey) and the study designs to be tested (binary variable: study type 1; study type 2) as independent variables. The trend was estimated using the survey date variable and the method effect using the study design variable. To estimate the trend as accurately as possible based on the available data, three options were tested: a linear fit for a straight line, a quadratic fit for a U-shaped or flattening trend and natural cubic splines for complex trends (an approximation of the trend using piecewise polynomials of degree three). For each indicator, the best fit to the data points was selected using the Bayesian information criterion [40]. For indicators that were only available at three time points, a linear trend was always used; as was the case where a study type was only available once across all time points. The models were established for the entire group and for subgroups stratified by gender identity, age, and education. In an additional analysis, stratification according to education was limited to the 30 – 64 age group, as the proportion of people with low education is higher among young adults (whose educational careers are not yet complete) and among those aged 80 and over.

Based on the results of the logistic regression analysis, the expected prevalence of the respective indicator can be predicted for studies of types 1 and 2 at each time point during the observation period. The method effect was then calculated as the difference between the predicted prevalences for the two study types:

Method effect =

*predicted prevalence for study type 1*

minus

*predicted prevalence for study type 2*

The models are based on the central assumption that the method effect remains constant over time. In logistic regression, the method effect is modelled using the odds ratio (OR), which is assumed to be constant over time. As the OR is difficult to interpret, we calculate the method effect as a difference instead, as described above. This difference can be determined at each time point and varies slightly over time, since logistic regression models the OR and not the difference as constant. The method effect is therefore defined as the difference at the time of the method change took place (August 2024, the median participation time of RKI Panel 2024).

In the figures, the observed prevalences from study type 2 (telephone sample with telephone interview) were additionally corrected for the method effect, i.e. the prevalences were adjusted to study type 1 (registry sample with written survey) by adding the method effect to the prevalence from study type 2. The confidence interval limits were converted in the same way.

The analysis included persons aged 18 and older. Data set version v3 was used for the recruitment study, and version v5 was used for the RKI Panel 2024. Trend analyses were weighted and, for the registry samples (study type 1), took clustering in the sample points into account using SAS 9.4 survey procedures (SAS Institute, Cary, NC, USA). Method effects (differences) were calculated with a 95 % confidence interval using the NLMeans macro in SAS. Each survey was weighted separately. To make the data sets from the different survey years comparable in terms of age and gender distribution, the weighting in each survey was adjusted to the European Standard Population [38]. For the stratified analyses by education level, standardisation was also carried out within each education group so that these analyses were based on the same age and gender distribution.

## 3. Results

### 3.1 Sample composition of the parallel surveys in 2024

#### Sample composition

To compare the two types of studies, we first examine the composition of the samples in the two parallel surveys: the GEDA 2024 telephone survey and the first two sub-waves (Q2 and Q3) of the RKI Panel 2024 (Table 2). Looking at the unweighted sample composition, shows that significantly more young people up to the age of 44 are represented in RKI Panel 2024 than in GEDA 2024. This group corresponds almost exactly to its proportion of the population in the RKI Panel 2024. People with low levels of education were also better represented in the RKI Panel 2024, although they remain underrepresented (19.3 % compared to 33.4 % in the Microcensus, and 14.2 % in GEDA 2024). The better representation of population shares by education level is evident in all age groups. In addition, the proportion of individuals living alone in the RKI Panel 2024 (22.3 %) is more consistent with the proportion recorded in the Microcensus (25.4 % of adults) than in GEDA 2024 (34.1 %). This finding is also evident within age groups, with the discrepancies becoming more pronounced with increasing age. The proportion of missing values in the variables characterising the sample composition is negligible in both studies (maximum value 0.4 %).

The weighted proportions by gender and age are almost identical in both studies, because both were weighted according to the same population distribution. However, differences can be seen in the weighted distributions of education and household size because GEDA and the RKI Panel were weighted according to different education classifications and household size was considered in the GEDA weighting. If GEDA 2024 were to be weighted in the same way as the RKI Panel (Table 2, row 4), the weighted proportions of education groups and of people living alone would also become similar.

### 3.2 Prevalence comparison of health indicators in the 2024 parallel surveys

Table 3 shows the prevalence of health indicators in the 2024 parallel surveys. Only weighted prevalences are shown here, to correct for differences in sample composition where possible through weighting. For this reason, the analyses stratified by education show the prevalences with age- and gender-standardisation within the education groups.

There is little difference in the prevalence rates of the indicators for general health status (*self-rated general health* and *chronic conditions*) between the two types of study. The age group 80 years and older is notable for both indicators. Here, RKI Panel 2024 shows a lower proportion of people reporting very good or good general health (not significant), and a significantly higher proportion of people with chronic conditions compared to GEDA 2024.

The prevalence estimates for mental health indicators reveal significant differences between the two parallel surveys. The prevalence of depressive symptoms is higher in RKI Panel 2024 than in GEDA 2024 in total and in almost all stratification groups. These differences are greater for women than for men (approximately 8 % versus 3 %). The differences are most pronounced in younger age groups (approximately 16 % for 18- to 29-year-olds and 6 % for 30- to 44-year-olds), whereas there are only minor or no differences in older age groups. The prevalence is higher in the RKI Panel 2024 than in GEDA 2024 in all education groups, with a more pronounced effect in the low education group, albeit with wide confidence intervals in this group. The estimates are also higher in the RKI Panel 2024 for anxiety symptoms, but less pronouncedly. These differences mainly occur among women (approx. 4 % difference) and 18- to 29-year-olds (approx. 11 % difference). These differences are primarily found in the medium and high education groups (approx. 3 % difference).

The self-rated mental health indicator showed a higher proportion of people with excellent or very good mental health in the RKI Panel 2024 compared to GEDA 2024. As this result contradicts the above findings on mental health and necessitates an adjustment to the analysis, a possible explanation is presented below. When we compare the prevalence rates from the RKI Panel 2024 the annual survey with the corresponding rates from the recruitment study conducted just a few months earlier, we find that the same individuals rate their mental health significantly worse in the recruitment study than in the annual survey. Even when limiting the analysis to individuals who completed questionnaire C in the first sub-wave of the annual survey (quarter 2), who were therefore asked the same question again within a short period of time (83 days on average) after the recruitment study, the difference in the proportion rating their mental health as excellent or very good was 45 % in the RKI Panel 2024 versus 34 % in the panel recruitment study. The positioning of the question in the questionnaire was identified as a possible explanation for these large differences. In the short recruitment study comprising 26 questions, this question appeared in position 16, after questions on general health and health behaviour. In Questionnaire C of the RKI Panel 2024, the question appeared later, in position 24, after questions about depressive symptoms, fatigue, and suicidal thoughts. In this context, participants apparently assessed their own situation as less negative. Due to this contextual or questionnaire effect, data from RKI Panel 2024 annual survey are not comparable with previous GEDA study results, in which this question introduced the mental health section. This effect appears to increase with age, as the deviations from the prevalences in the recruitment study increase with age. Therefore, in the trend analyses described in the following section, data from the panel’s recruitment study is used for this indicator instead of data from the annual survey.

Obesity prevalence in the RKI Panel 2024 is three percentage points higher than in the GEDA 2024 survey. The effect is slightly stronger among women and more pronounced among 18- to 29-year-olds (a difference of more than 6 percentage points). Conversely, smoking prevalence, is more than three percentage points lower in the RKI Panel 2024 than in GEDA 2024, with more pronounced differences among men, middle-aged individuals and those with middle-level education.

In sensitivity analyses analogous to the RKI Panel, the weighting of GEDA 2024 had little effect on the prevalence estimates. The change was mostly between zero and a maximum of two percentage points, tending to increase the size of the method effects. For the smoking indicator only, the method effect decreased by about two percentage points as a result of the changed weighting, disappearing completely for women.

### 3.3 Response behaviour depending on the survey mode in the parallel surveys 2024

This section describes patterns in response behaviour resulting from changes in survey mode for different question and answer formats, such as categorical or metric answers, and simple or multiple Likert scales. Additionally, it describes the proportions of missing values depending on the response format.

#### Response format: Single Likert scale

The two indicators, *self-rated general health* and *self-rated mental health*, are each based on a single Likert scale with five response options. The first two options are combined for each indicator.

As described in the previous section, the indicator of *self-rated general health* (‘very good’ or ‘good’) shows only minor differences in a parallel comparison, (64 % in the RKI Panel 2024 vs. 65 % in GEDA 2024, Table 3). However, examining the individual categories reveals shifts: in GEDA 2024, a higher proportion of people rated their health as ‘very good’ (19 %) than in RKI Panel 2024 (12 %), while ‘good’ is mentioned more frequently in RKI Panel (52 % vs. 46 %). With regard to the individual categories at the other end of the scale, marginal categories are selected less frequently and middle categories more frequently in the RKI Panel’s written survey.

Due to the contradictory results for the self-rated mental health indicator described above, which are probably due to sequence effects in the questionnaire, the response behaviour for this indicator is analysed using the identical question from the RKI Panel recruitment study. As with self-rated general health, there is hardly any difference when looking at the indicator with the combined categories (‘excellent’ or ‘very good’), at 25 % in GEDA 2024 versus 26 % in the RKI Panel recruitment study 2024. When examining the individual categories, the RKI Panel 2024 once again exhibits the expected response pattern for written surveys, with lower frequencies in the marginal categories and higher frequencies in the middle categories. The category ‘excellent’ was selected less frequently in the written survey (8.1 % vs. 11.7 %), while ‘very good’ was selected more frequently (17.9 % vs. 13.2 %).

#### Response format: multiple Likert scales

The indicators for *depressive symptoms* and *anxiety symptoms* are based on eight and two items, respectively. They use a Likert scale response format and values are assigned to each answer category. These values are added together to give a total score, which is then classified on the basis of a fixed threshold value.

**Figure 1:**
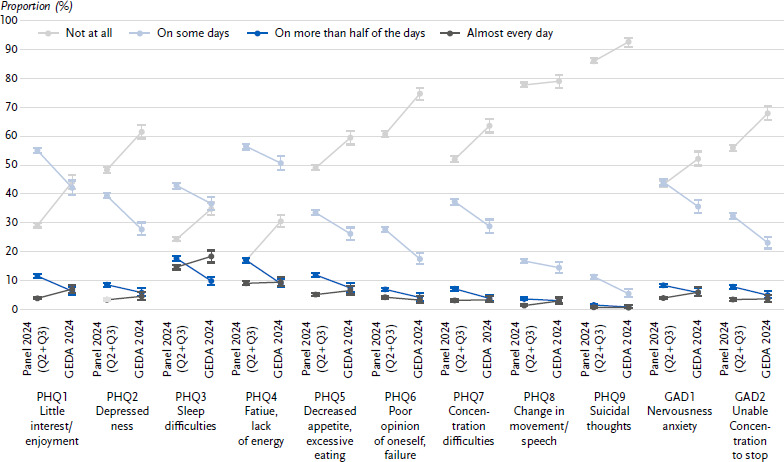
Shift in the proportions across the individual response categories for each of the eight items in the PHQ-8 (depressive symptoms and the two items in the GAD-2 (anxiety symptoms) in the parallel surveys in Q2 and Q3 of 2024 (weighted analyses); grey categories represent the marginal categories; blue categories represent the middle categories. Source: Parallel Surveys GEDA 2024 and RKI Panel 2024 (Q2 + Q3) PHQ-8 = Patient Health Questionnaire-8, GAD-2 = Generalised Anxiety Disorder-2

Clear differences can be seen between GEDA 2024 and the RKI Panel 2024 in the indicator of depressive symptoms (see the prevalence comparison in Table 3), which is based on eight individual items (PHQ1 to PHQ8). For all individual items, the RKI Panel shows a shift towards the middle response options compared to the telephone survey (Figure 1). The extreme categories ‘Not at all’ and ‘Almost every day’ (shown in grey in Figure 1) are selected less frequently in the RKI Panel, while the middle categories ‘On some days’ and ‘On more than half of the days’ are selected more frequently (shown in blue). As the category ‘Not at all’ is mentioned significantly more often than ‘Almost every day’ in both surveys, the observed shift in the RKI Panel 2024 leads to higher total values. Consequently, the prevalence of depressive symptoms is higher in the RKI Panel 2024 than in GEDA 2024.

The indicator of anxiety symptoms is based on two items (GAD1 and GAD2), which show the same pattern as the eight individual depressive symptom items (Figure 1). Again, the middle categories were reported more frequently in the written survey than in the telephone. Since only two items are added up, the effect on the prevalence estimators for anxiety symptoms is smaller than for depressive symptoms (Table 3).

#### Response format: categorisation of metric data

The *obesity* indicator is based on self-reported metric data on height and weight, from which BMI is calculated. The height data does not differ between the two surveys. However, the body weight data is slightly higher on average in Panel 2024 than in GEDA 2024 (79.9 kg versus 78.3 kg). The percentiles are also higher in Panel 2024 than in GEDA 2024 (e.g. 90th percentile 104 kg vs. 100 kg or 95th percentile 115 kg versus 110 kg). These relatively small changes in average weight, lead to higher BMI values and result in a non-negligible three percentage point difference in obesity prevalence due to BMI categorisation using the fixed threshold of ≥ 30 kg/m^2^.

#### Response format: Categorical

For the *chronic conditions* indicator, which is based on a simple yes/no question, there are no differences in response behaviour between the survey modes (Table 3).

The smoking indicator is based on a categorical question with four possible answers. The lower prevalence of current smokers (‘yes, daily’ or ‘yes, occasionally’) in RKI Panel 2024 compared to GEDA 2024 is mainly due to a lower proportion of respondents in the ‘yes, daily’ category (18.0 % vs. 20.9 %) and a higher proportion of respondents who stated that they had never smoked (46.6 % vs. 44.2 %), representing the ’never smoked’ category. The middle categories (occasional or former smokers) show similar prevalences in the two surveys. There is no discernible shift towards the marginal categories, as would usually be expected in telephone surveys where the first and last categories are often reported more frequently.

#### Proportion of missing values depending on the response format

Table 3 shows the proportions of missing values. In general, the proportions are very low in GEDA 2024 and slightly higher in RKI Panel 2024, mainly due to paper questionnaires being used for the panel. However, even in online mode, there are slightly higher proportions of missing values than in telephone interviews. This applies, for example, to the two indicators of general health status (*self-rated general health*, *chronic conditions*), for which a simple Likert scale or categorical variable is used. In all subgroups and both studies, the proportion of missing values is a maximum of 1 %, and is significantly lower in most cases. An exception is the elderly (aged 80 and older), who have around 3 % missing values for both indicators in the RKI Panel (compared to 1 % and 0 % in GEDA 2024). This is due to the high proportion of paper questionnaires in this age group. For the categorical indicator *smoking*, there are almost no missing values in telephone interviews in GEDA 2024, but 1.7 % in the overall group in RKI Panel 2024. This proportion is slightly higher in older age groups and among those with lower levels of education (up to 8 % and just under 3 %, respectively).

In the case of multiple Likert scales (e.g. *depressive symptoms* and *anxiety symptoms*), the proportion of missing values increases with the number of underlying questions, since the sum values are only calculated if all the individual questions have been answered. For anxiety symptoms (two items), the proportion of missing values is mostly in the range of 1 to 2 %, with no clear differences between study types. For depressive symptoms (eight items), 2.4 % of values are missing in the RKI Panel 2024 for the overall group. In contrast, in GEDA 2024, the proportion is significantly higher at 4.4 %. This difference between study types is evident in all subgroups. For both indicators, in both study types, the proportion of missing values is significantly higher in older age groups and in the low education group than in their respective comparison groups.

Higher proportions of missing values were also found in the telephone interview in GEDA 2024 (2.7 %) than in the RKI Panel 2024 (1.1 %) are also found for obesity. The difference between the studies is most pronounced among women (4.2 % vs. 1.4 %) and young adults (3.2 % vs. 0.9 %).

### 3.4 Method effect in analyses over time

The following age- and gender-standardised analysis includes the full RKI survey time series from 2003 to 2024. As well as the annual RKI Panel survey in 2024 (here without restriction to quarters 2 and 3), it takes into account data from the recruitment study. The aim is to assess the prevalence trend over time, while controlling for the method-related effects.

**Figure 2:**
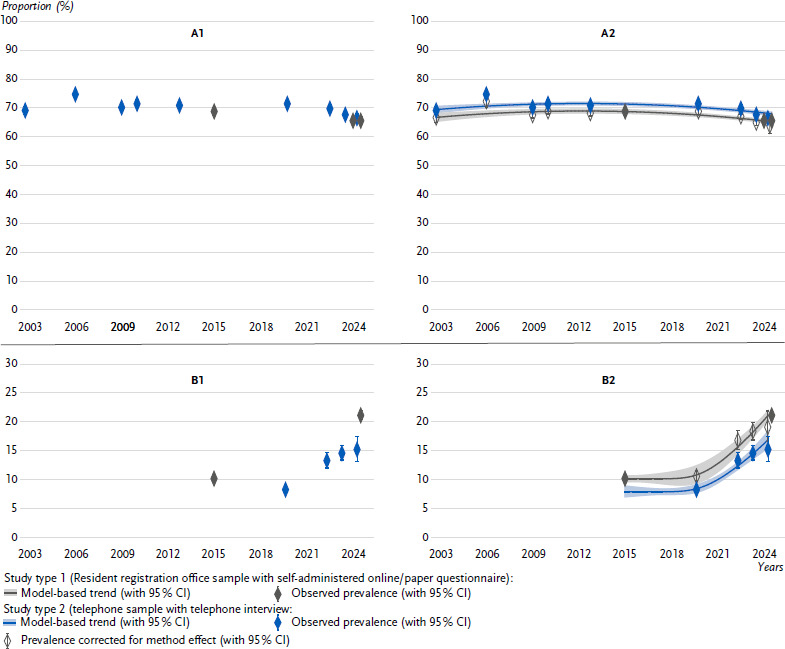
Presentation of prevalence development over time (2003–2024) taking into account the method effect for two exemplary indicators (analysis for the total population without stratification). Source: RKI health surveys (GSTel, GEDA), recruitment study for the RKI Panel 2024, GEDA 2024, RKI Panel 2024. A: Indicator: self-rated general health; B: Indicator: depressive symptoms A1 and B1: Prevalence estimates from the various surveys A2 and B2: Additionally: estimated trend for study type 1 and study type 2, respectively

Figure 2 shows the results of the model-based time series analysis for two example indicators. The complete time series analyses, including stratification, are presented for various indicators in indicator sheets published on the RKI publication server (https://edoc.rki.de/). Example A (*self-rated general health*) shows an indicator with a large number of data points, three of which are from study type 1: one in the middle and two at the end of the observation period. This allows for a relatively robust estimate of the method effect. The proportion of adults in very good or good general health has declined slightly in telephone samples in recent years, particularly following the period of the COVID-19 pandemic (from 69.0 % in GSTel03 to 69.6 % in GEDA 2022 and 67.5 % in GEDA 2023). The registry samples also show a downward trend at a slightly lower level: from 68.7 % in GEDA 2014/2015-EHIS to 65.4 % in Panel 2024. The method effect, i.e. the difference in prevalence between the two study types, is moderate at - 2.8 percentage points and indicates lower prevalences in the registry samples. The parallel trend estimated by the model for the two study types fits well with the individual studies’ prevalence estimates, justifying the assumption of a constant method effect over time. Comparing GEDA 2023 with Panel 2024 directly shows a decrease in prevalence of 2.1 percentage points. However, if we look at the trend lines from 2023 to 2024, corrected for the method effect, the downward trend is estimated at only 0.6 percentage points. In other words, the temporal trend would be overestimated without modelling the method effect.

Example B (*depressive symptoms*) has fewer data points, but two surveys of study type 1 are also located at either end of the observation period here. The prevalences show an increasing proportion of depressive symptoms since GEDA 2014/2015-EHIS, again with a significant increase after the pandemic, which cannot be optimally represented, even by the flexible spline model. Nevertheless, it seems obvious that there is a relatively large method effect of about 4 percentage points. A direct comparison of GEDA 2023 (14.6 %) with Panel 2024 (21.2 %) reveals an increase of almost 7 percentage points, while the model corrected for the method effect predicts an increase of only 2 to 3 percentage points.

Table 4 provides an overview of the estimated method effects, expressed as the difference between the two trend lines. The method effects are stronger for the indicators of mental health, social support and smoking prevalence than for physical health and obesity indicators.

For mental health indicators, method effects in analyses over time are more pronounced in women than in men and decrease with age (exception: method effect for the anxiety symptoms indicator in the age group 80 years and older). Overall, particularly strong effects are evident for the age group 18 to 29. For example, the effect of change in methods on the prevalence of depressive symptoms is estimated at 11 percentage points in this age group.

In terms of physical health indicators, there are hardly any differences between the sexes method effects over time. The age group 80 years and older shows the greatest method effects for almost all physical health indicators, and these effects exceed those on mental health in the same age group. For the obesity indicator, there are only minor method effects between the two study types over time, and these hardly differ between subgroups. However, the effects of smoking are more pronounced and significantly greater for men than for women.

When education groups are compared, the method effects do not initially present a consistent picture across the indicators. Therefore, an additional analysis was limited to the age groups between 30 and 64 years, as these are more meaningful in terms of educational differences. Indicators of both physical and psychosocial health show that the highly educated group in the registry samples tends to report complaints more frequently than the highly educated group in the telephone samples. Conversely, the low educated group in registry samples tends to report complaints less frequently, suggesting smaller method effects. The exceptions are smoking and obesity risk factor indicators, social support, and self-rated general health.

## 4. Discussion

With the launch of the RKI Panel ‘Health in Germany’, the previously conducted GEDA health monitoring studies, which were primarily conducted by telephone, were replaced with a self-administered, mixed-mode design (paper and online) based on a registry sample. The change in methodology aims to improve data quality and facilitates long-term data collection, enabling reliable mapping of trends in key health indicators for Germany based on data. To ensure comparability with previous surveys, a parallel survey with identical content but different survey mode was conducted. The sample composition, prevalence estimates and response behaviour of both studies were compared and method-related differences were quantified. In addition, a model was presented that can be used to estimate trends while controlling for the method effect.

###  

#### Method effects in sample composition

The comparison of the parallel surveys in 2024 suggests the impact of the switch from the telephone dual-frame design of the GEDA studies to a self-administered mixed-mode survey based on a population register sample on the composition of the sample. The RKI ‘Health in Germany’ Panel provides a more accurate representation of the German population: young adults, people with low levels of education, single-person households, the elderly and people with health restrictions are better represented than in previous telephone studies.

The results confirm the methodological advantages of population register samples over telephone samples, as described in the literature [28, 41]. The more complete coverage of the population, the possibility of stratified random selection with known selection probabilities and the independence from the target person’s telephone availability or equipment reduce selection bias, contributing to less biased overall sample [41]. Against the backdrop of declining participation rates, which are particularly pronounced in telephone samples [7], the switch to a mixed-mode design seems a logical and timely move.

Current studies also show that mixed-mode surveys in Germany exhibit the least education bias, while self-administered online surveys and, particularly telephone surveys, exhibit the most bias [6]. The use of multiple survey modes is therefore a key strategy for reducing systematic errors [14, 15, 42]. Using different access channels in parallel can reduce differences between respondents and non-respondents, thereby mitigating non-response bias. Taking different preferences for digital technologies into account also contributes to this. While older people are more likely to use paper questionnaires [12, 43], younger and more highly educated groups prefer digital devices [44, 45]. Incentives are also likely to have contributed to improved representation. The unconditional remuneration in the recruitment study and the conditional bonus for registering in the RKI Panel may have been an effective incentive to participate, especially for younger and lower-income individuals.

The targeted over-recruitment of certain age groups, particularly those aged 80 and older, also helped to improve the representation of these population groups [23]. The higher prevalence of chronic conditions and impairments in physical function observed in this age group suggests improved accessibility for people with health impairments, as previous methodological studies have also shown [46].

At the same time, new selection mechanisms may emerge. Panel surveys require long-term participation, which may particularly appeal to health-conscious and health-impaired individuals. Similar trends have been observed in other surveys, such as the overrepresentation of people with mental health problems in non-probability online panels (panels comprising individuals who have registered of their own interest) [14]. The lower smoking prevalence in the RKI Panel 2024 could indicate disproportionate participation by health-conscious individuals, although part of this difference is due to changes in weighting. It is possible that a selection of health-conscious individuals occurred during recruitment, whereas participation in telephone surveys is more strongly influenced by situational factors. This is supported by the finding of other studies that there is no significant difference in smoking prevalence between written and telephone surveys (e.g. [47]) – in some cases, telephone interviews have even reported more favourable health behaviour [12].

The overall higher prevalence of psychological distress and lower social support, as well as the higher proportion of chronically ill and physically disabled people among the elderly indicate improved representation of health-vulnerable groups. Nevertheless, it remains unclear whether these groups are proportionally represented within the population. Initial findings suggest that people with health problems may be slightly less likely to re-participate [23], which could also have affected initial participation in the RKI Panel.

#### Method effects on prevalence estimates and response behaviour

A comparison of the two parallel studies shows significant differences in prevalence between the two study designs. Mental health indicators such as depressive and anxiety symptoms or low social support are significantly higher in the RKI Panel than in the GEDA telephone survey, especially among women and younger adults. The differences are less pronounced for physical health indicators; obesity is moderately more prevalent, while smoking is slightly less prevalent. These deviations should primarily be viewed in the context of the switch from telephone interviews to a self-administered mixed-mode survey and can be explained by the effects of the survey mode and differences in questionnaire design. Only in the case of smoking does the change in weighting play a role.

Depending on the survey mode, respondents may provide different answers to the same question [14, 42, 48], which can lead to systematic distortions, known as mode effects (e.g. [14, 49]). The sensitivity of the topic, the wording and design of questions and the order in which they are presented, as well as the format of the response options, also influence response behaviour [27]. In line with previous studies (e.g. [12, 39]), the greatest deviations are found in subjective assessments and Likert scales, such as those used to measure mental health indicators (e.g. depressive symptoms). Likert scales are particularly susceptible to mode-dependent response patterns [26, 39], as cognitive processes and satisficing (making simplified responses without fully considering each question) may vary depending on the survey mode due to different levels of respondent burden [28, 48]. Written surveys which are processed visually, often lead to primacy effects (selecting the first category read) [27, 48], less extreme responses [12] and a stronger tendency towards the middle of the scale, i.e. a neutral or middle category [26]. This tendency towards the middle is particularly pronounced when the scale has a clear middle category [26, 27]. Conversely, telephone interviews, which appeal to the sense of hearing, favour recency effects, i.e. the choice of the last answer option heard [27, 28, 48, 50]. Depending on the order of the answer options, such effects can accumulate and alter the sum values. The categorisation of the sum values required for prevalence estimates can further amplify these effects [51].

Another key influencing factor is the extent of socially desirable response behaviour, which varies with the perceived privacy of the survey. In telephone or face-to-face interviews, interviewer effects can lead to socially desirable responses, rounding up (heaping), extremely positive ratings and acquiescence [14, 19, 26–28, 42, 48]. When in the presence of other, respondents tend to present themselves in a more favourable light, give answers that align with social norms, and offer more positive information overall [9, 26–28, 47–49, 52, 53]. Socially desirable content tends to be overreported, while undesirable content is underreported, so that interview-based health surveys tend to overestimate positive health status and health-promoting behaviours [28]. In self-administered surveys, however, responses tend to be more realistic, especially regarding sensitive topics [9, 14, 28, 52]. As has already been observed for other psychometric inventories or single-item measures [12, 48, 54], this pattern, in addition to cognitive processing, may help explain why indicators of mental health in the RKI Panel tend to show higher levels of psychological burden. Studies on the measurement invariance of psychometric scales demonstrate that while different survey methods measure the same constructs, mean values between the survey modes differ systematically, typically in association with socially desirable response behaviour [42, 48]. The higher proportion of missing data in the two screening inventories for depressive and anxiety symptoms suggests that multiple Likert scales are more difficult to answer over the telephone and that, social desirability or shame may also play a role as with the weight data required for calculating obesity prevalence.

In contrast, objective dichotomous items, such as the indication of a diabetes diagnosis, show no systematic effects of the survey mode [see also 12, 19, 39]. Metric data, such as body weight, more often contain minor systematic deviations. In written questionnaires, participants often report a slightly higher weight [55], possibly because they weigh themselves in advance and give less socially desirable answers. This effect can be exacerbated by heaping effects such as rounding up or down in telephone interviews [56]. Even small differences can change the prevalence of obesity in BMI categories by several percentage points [57] and influence group comparisons, a well-known effect of categorisation [51].

According to meta-analyses, there are hardly any differences in response behaviour between self-administered written modes, i.e. online and paper questionnaires [28, 58]. Written surveys allow for more thorough cognitive processing of the questions as there is less time pressure and information can be checked or interrupted [15]. The tendency to select the middle option in written surveys could also reflect more differentiated cognitive processing and more reflective responses, providing more valid information.

An apparent contradiction arises with regard to the indicator of self-rated mental health. In the RKI Panel 2024 annual survey, more respondents reported very good or excellent mental health, despite other indicators showing higher levels of psychological stress. The comparison with the recruitment study, where the same question was asked in a different context, suggests that the order of questions in the questionnaire influence responses (see Methods section). This finding demonstrates that seemingly minor methodological changes, such as placement of a question within a questionnaire, can systematically influence response behaviour [27]. This emphasises the importance of careful questionnaire design and thorough testing in advance to minimise systematic errors (bias) [49].

In addition to classic mode effects, satisficing processes could also play a role, i.e. the tendency to answer cognitively demanding questions with minimal effort [59]. This primarily occurs in long, complex or stressful questionnaires [26], such as those used in the field of mental health. Many mental health items are similar in content and concern sensitive topics, which could lead to fatigue or the desire to complete the questionnaire quickly. However, initial analyses show no evidence of increased item non-response, dropout rates or straightlining (always selecting the same response category across multiple questions). An in-depth review, including initial data from the RKI Panel 2025, is currently underway.

It is also possible that thematic division of questions across several questionnaires influences prevalences if people with corresponding symptoms participate more in the-matically appropriate modules. For example, specific pain surveys have been reported to lead to higher pain prevalence rates than surveys covering a comprehensive range of health topics [60, 61].

#### Different method effects in different population groups

The analyses show that method-related effects are not evenly distributed across all population groups. Over time, these effects tend to be more pronounced in women than in men, except for indicators relating to long-term activity limitations, diabetes and smoking. The differences are particularly evident among young adults, especially with regard to mental health indicators. This age group is better represented in the RKI Panel 2024, indicating both improved sample coverage and a change in response behaviour. However, younger people show the least participation stability, as evidenced by the declining participation rates across the sub-waves of the RKI Panel 2024 [18]. It cannot be ruled out that a selection has occurred within this age group, e.g. through a higher re-participation among people interested in health or those who are particularly burdened.

Among 80-year-olds and older, the greatest method effects occur in the area of physical health. It appears that more severely disabled individuals were reached in this group, as reflected by higher prevalences of chronic diseases and disabilities in the RKI Panel 2024. This finding is consistent with earlier studies [46, 62], which emphasize that more complex recruitment and survey designs are required to include the very elderly and the physically disabled, as demonstrated in the ‘Health 65 +’ study for nationwide monitoring [63].

When comparing education groups, limiting the analysis to the age group 30 to 64 years in the self-administered registry sample survey revealed that higher education was associated with a higher prevalence of complaints, while lower education was associated with lower prevalence rates. Without age restrictions, however, these method effects specific to education overlap with age effects, given that the proportion of people with low education is particularly high among 80-year-olds and older and younger people who have not yet completed their education. The fact that this pattern is evident for both physical and mental health, as well as and for factual and attitudinal indicators, suggests that, the registry samples generally included more healthy people with low levels of education and more people with poorer health among those with higher levels of education. It is also possible that there is an education-dependent response behaviour, whereby either fewer or more complaints are reported depending on the study design. The education gradient of the method effect is particularly strong for psychosocial health indicators. One possible explanation for this could be the stronger effects of social desirability among people with higher education, as demonstrated by indicators of well-being [64]. The higher estimated prevalence of poor health among people with higher education and the lower prevalence among those with lower education suggests that the educational differences observed in the RKI Panel may be less pronounced than in previous telephone surveys.

The results show that method effects can influence both the overall prevalence level and the extent of estimated health inequalities. Differences in prevalence estimates cannot be clearly attributed to changes in sample composition or response behaviour. Therefore, the estimated value encompasses all changes associated with the study design.

#### Estimation of method effects

Taking the study type into account when modelling the time series enabled the simultaneous estimation of trend and method-related effects. This revealed that these effects can be significant, sometimes exceeding the change overserved over time in individual cases and generally exceeding the random error [65]. Without taking these effects into account could lead to apparent trend breaks being misinterpreted as actual changes in health.

A key assumption in the modelling is that the method effect remains over time. This assumption has proven plausible for most indicators due to the good fit of the modelled curves to the given data points (see also 3.4). This makes it possible to draw conclusions about prevalence trends over time despite changes in survey methods, albeit with greater uncertainty. However, for indicators with few data points for one of the two study types or at the edges of the time series, greater uncertainty remains, so supplementary sensitivity analyses and cautious interpretation are advisable in these cases.

In addition to the data situation, it is also necessary to examine whether it is justified to assume a constant method effect for the respective indicator. The time series of the RKI surveys goes back up to 20 years. During this period, social attitudes may have shifted which could have affected the extend of social desirability. In the fast-paced world of social media, acceptance of topics and attitudes can change within a relatively short period of time. The declining participation rates in the telephone surveys ([7], Annex Table 1) may influence sample composition and thus differences to registry based studies. The increasing acceptance of online questionnaires, even among older age groups, may shift the proportions of individual survey methods in the mixed-mode design over time, thereby affecting prevalence estimates. Therefore, the assumption of a constant method effect always represents a certain simplification. Nevertheless, if supported by the data, it can be helpful, in estimating the order of magnitude of method effects.

#### Strengths and limitations

Method effects are not a singular phenomenon of the RKI health surveys but affect all studies based on voluntary participation. However, the launch of the RKI Health Panel enabled the method effect to be estimated and at the development of the prevalences of interest to be assessed over time. This was achieved by conducting a parallel survey with a design analogous to previous studies and modelling the entire time series from two different study designs. A key prerequisite for this approach was the use of a comparable design to that of the RKI Panel in an earlier survey in 2014/2015. However, as there is only one previous data point with this design, the estimated method effects should be understood as an approximation of the effects of the change in methodology, assuming a constant method effect over time.

The analyses suggest that samples from resident registration office with self-administered surveys, such as the RKI Panel 2024, more realistically reflect the population structure than telephone surveys. Nevertheless, sources of bias remain, particularly with regard to lower participation rates among people with lower levels of education. However, the weighting could only account for the basic socio-demographic characteristics of age, gender, region, type of municipality, education and household size. Furthermore, no systematic comparison with other external reference data was carried out. Therefore, potential biases, such as those commonly occurring in cross-sectional studies, related to political attitudes, health awareness or motivation to participate, cannot be ruled out.

As with the telephone surveys, the RKI Panel 2024 only used German-language survey instruments. Consequently, individuals for whom German is not their preferred language cannot be adequately represented. To counteract this bias in future, we are considering the use of selected translated materials and questionnaires for future survey waves and, in particular, recruitment studies.

Since the studies conducted in parallel are not based on the same individuals, changes in response behaviour can only be assessed by comparing the frequency of response categories, rather than intra-individually. Furthermore, only a few measurement points were available for some indicators to date, which limits the robustness of the modelling. Overall, even for longer time series, there were only a few data points based on registry samples with written surveys. Especially at the edges of the observation period, trends can only be approximated to a limited extent. Future surveys will therefore be crucial in confirming or refuting observed changes in trend.

The presented method enables to quantify the method effect for each indicator and thus to compare prevalence estimates from different study designs. This provides a foundation for classifying prevalence estimates from different study designs, extending beyond RKI studies.

#### Conclusions

The establishment of a panel infrastructure with predominantly cost-effective online surveys enables methodologically consistent data collection for health monitoring in the future. This is a basic prerequisite for valid trend statements [11–13]. The RKI Panel 2024 sample reflects population structure in terms of age, gender, household size, and education level more accurately than the earlier GEDA studies did. Initial indications suggest that individuals with poorer health or increased psychological stress are more likely to participate than in telephone surveys, in which they tend to be underrepresented. In contrast, smoking behaviour indicates a slightly higher participation rate among health-conscious individuals, which could introduce bias. The overall impact of these effects cannot be conclusively assessed at this time.

Compared to cross-sectional surveys, the panel design presents the challenge of attrition, whereby participants systematically dropout of over time. This can be counteracted through targeted participation incentives, panel maintenance, weighting corrections, and refreshment samples. Nevertheless, given the decline in participation rates in population surveys (e.g. [8]), this remains a key challenge in terms of data quality and representativeness.

As already observed in the methodological study on the change of methodology to GEDA 2014/2015 [19], the self-administered mixed-mode design (paper and online) used in the RKI Panel can lead to more valid data due to the elimination of interviewer effects and greater privacy, especially for sensitive questions [14, 58]. Written surveys also offer flexibility in terms of time, as respondents can interrupt, reconsider or research their responses. At the same time, this increases the demands on questionnaire design and dramaturgy. As the same people are repeatedly surveyed, a reasonable survey length [66], clear structure and high comprehensibility are crucial to avoid satisficing. Careful pretesting, regular satisfaction surveys and appropriate incentives for participation are therefore important components of quality assurance.

The analyses highlight the importance of maintaining methodological consistency over time, in terms of both study design (sampling, survey method) and measurement instruments (question and answer wording, position in the questionnaire). Where changes are unavoidable, they should be accompanied by methodological studies to quantify possible effects. This applies in particular to mental health indicators, which are susceptible to mode and context effects. Only in this way can observed differences be interpreted as substantive changes, rather than measurement artefacts. The statistical methods employed here enabled method-related effects to be identified and estimated.

## Figures and Tables

**Table 1:** Overview of the data bases for the analysed indicators

Survey	Sampling		Survey mode	Indicator										
	Residents’ registration office (registry) sample	Telephone sample: landline (L)/dual frame (DF)	Mixed mode written (web-based 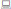 /paper 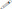 )/Telephone interview 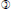	Self-rated general health	Chronic conditions	Long-term activity limitations	Impairments in physical function	Diabetes mellitus	Obesity	Smoking	Self-rated mental health	Depressive symptoms	Anxiety symptoms	Social support
GSTel03 (2003)		L	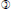	X				X	X	X				
GSTel06 (2006)		L	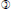	X				X	X	X				
GEDA 2009		L	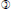	X					X	X				
GEDA 2010		L	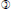	X				X	X	X				X
GEDA 2012		L	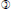	X				X	X	X				X
GEDA 2014/2015-EHIS	Registry		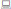 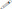	X	X		X		X	X		X		X
GEDA 2019/2020-EHIS		DF	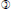	X	X	X	X		X	X		X		X
GEDA 2022		DF	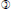	X	X	X		X	X	X	X	X	X	X
GEDA 2023		DF	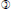	X	X	X			X	X	X	X	X	X
RKI-Panel 2024 Recruitment Study	Registry		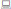 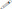	X	X				X	X	X			
GEDA 2024		DF	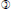	X	X				X	X	X	X	X	
RKI-Panel 2024	Registry		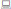 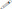	X	X	X	X	X	X	(X)^*^	X	X	X	X

Notes: Registry = Residents’ Registration Office, DF = Dual Frame = selection based on landline and mobile phone, GSTel = Telephone Health Survey, GEDA = German Health Update, EHIS = European Health Interview Survey.

^°^The question about smoking was only asked to people without diabetes in the RKI Panel 2024 in order to calculate a diabetes risk score. For people with diabetes, the information on smoking status was therefore taken from the recruitment study.

**Table 2:** Sample composition in the parallel surveys for the second and third quarters of 2024 (row percentages and 95 % confidence intervals)

Survey and study type		Gender identity		Age group (years)					Education group			Household size°	
		Female	Male	18 – 29	30 – 44	45 – 64	65 – 79	≥ 80	Low	Medium	High	Single-person household	Multi-person household
Proportions according to the Federal Statistical Office^*^	%	51.1	48.9	16.0	23.5	33.7	18.1	8.7	33.4	46.6	20.0	25.4	74.6
Study type 1: RKI Panel 2024 (Q2 + Q3) (n = 18,222)	n	9,853	8,324	2,641	3,894	6,035	4,150	1,502	3,519	8,706	5,965	4,062	14,157
	% unweighted	54.2 (53.5 – 54.9)	45.8 (45.1 – 46.5)	14.5 (13.9 – 15.1)	21.4 (20.6 – 22.2)	33.1 (32.3 – 33.9)	22.8 (22.1 – 23.5)	8.2 (7.8 – 8.7)	19.3 (18.4 – 20.3)	47.9 (46.8 – 49.0)	32.8 (31.3 – 34.4)	22.3 (21.4 – 23.2)	77.7 (76.8 – 78.6)
	% weighted	50.5 (49.6 – 51.5)	49.5 (48.5 – 50.4)	16.0 (15.2 – 16.7)	23.7 (22.9 – 24.5)	33.4 (32.6 – 34.2)	18.4 (17.9 – 19.0)	8.6 (8.1 – 9.1)	33.1 (31.7 – 34.6)	46.4 (45.2 – 47.7)	20.4 (19.2 – 21.7)	26.3 (25.3 – 27.4)	73.7 (72.6 – 74.7)
	Missing values	45 (0.2)		0 (0.0)					32 (0.2)			3 (0.0)	
Study type 2: GEDA 2024 (n = 4,016)	n	2,147	1,863	341	600	1,408	1,112	555	569	1,761	1,678	1,367	2,641
	% unweighted	53.5 (52.0 – 55.1)	46.5 (44.9 – 48.0)	8.5 (7.7 – 9.4)	14.9 (13.9 – 16.1)	35.1 (33.6 – 36.6)	27.7 (26.3 – 29.1)	13.8 (12.8 – 14.9)	14.2 (13.1 – 15.3)	43.9 (42.4 – 45.5)	41.9 (40.3 – 43.4)	34.1 (32.7 – 35.6)	65.9 (64.4 – 67.3)
	% weighted**	50.7 (48.3 – 53.2)	49.3 (46.8 – 51.7)	16.1 (14.1 – 18.3)	22.9 (20.7 – 25.4)	33.9 (31.6 – 36.2)	18.3 (16.7 – 20.0)	8.8 (7.7 – 10.1)	23.6 (21.3 – 26.0)	56.2 (53.8 – 58.6)	20.2 (18.8 – 21.6)	37.4 (34.9 – 39.9)	62.6 (60.1 – 65.1)
	% weighted analogously to RKI Panel***	51.0 (48.6 – 53.4)	49.0 (46.6 – 51.4)	15.9 (14.1 – 18.0)	22.3 (20.1 – 24.5)	34.2 (32.1 – 36.5)	18.8 (17.3 – 20.5)	8.7 (7.8 – 9.8)	31.8 (29.3 – 34.4)	48.0 (45.7 – 50.4)	20.2 (18.8 – 21.6)	25.7 (23.7 – 27.7)	74.3 (72.3 – 76.3)
	Missing values (n,% weight**)	6 (0.2)		0 (0.0)					8 (0.4)			8 (0.3)	
p-values	p-values (unweighted)	0.44		< 0.0001					< 0.0001			< 0.0001	
	p-values (weighted)**	0.87		0.97					< 0.0001			< 0.0001	
	p-values (weighted)***	0.72		0.75					0.47			0.56	

The data for the RKI Panel 2024 refers to participants who answered Questionnaire C; the values for participants who answered Questionnaire A are very similar.

°In the RKI Panel 2024, the information on household size was taken from the recruitment study if it was missing in the annual survey (e.g. due to non-participation in quarter 2).

*Distribution of gender and age: official population figures as of 31 December 2023; distribution of education group and household size: 2021 Microcensus

**GEDA 2024 with weighting analogous to previous GEDA studies (education weighting with ISCED, no weighting by household size)

***GEDA 2024 with weighting analogous to the RKI Panel (education weighting using CASMIN, with weighting by household size)

**Table 3:** Comparison of health indicators in the parallel surveys in quarters 2 and 3 of 2024 (weighted percentages and 95 % confidence intervals)

Indicator (number of valid values in RKI Panel 2024 / GEDA 2024)		Gender identity			Age group (years)					Education group		
		Total	Female	Male	18 – 29	30 – 44	45 – 64	65 – 79	≥ 80	Low	Medium	High
Self-rated general health Very good/good (n = 18,519/4,010)	RKI Panel 2024 (Q2 + Q3)	64.2 % (63.1 – 65.4)	61.5 % (60.0 – 63.0)	67.0 % (65.5 – 68.6)	78.3 % (75.7 – 80.7)	73.3 % (71.0 – 75.5)	63.2 % (61.4 – 64.9)	55.6 % (53.9 – 57.4)	35.2 % (31.9 – 38.5)	54.6 % (51.6 – 57.5)	67.5 % (66.1 – 68.8)	77.3 % (75.9 – 78.7)
	GEDA 2024	65.1 % (62.6 – 67.4)	65.4 % (62.0 – 68.6)	64.7 % (61.1 – 68.1)	82.9 % (76.8 – 87.7)	77.9 % (72.0 – 82.9)	60.6 % (56.5 – 64.5)	52.6 % (47.9 – 57.3)	42.0 % (35.1 – 49.2)	59.3 % (50.8 – 67.3)	65.4 % (61.9 – 68.7)	80.0 % (77.5 – 82.2)
	Missing values (n,% weighted)°	85 (0.6)	53 (0.8)	32 (0.4)	1 (0.2)	3 (0.2)	11 (0.2)	30 (0.8)	40 (3.2)	44 (0.9)	24 (0.3)	14 (0.3)
		6 (0.3)	4 (0.2)	2 (0.5)	0 (0.0)	1 (0.1)	1 (0.6)	0 (0.0)	4 (1.0)	2 (0.1)	2 (0.4)	2 (0.1)
	p-value*	0.53	0.56		0.46					0.72		
	p-value within groups		0.0423	0.22	0.1597	0.1443	0.24	0.24	0.0811	0.30	0.27	0.0638
Chronic conditions Yes (n = 18,513/4,006)	RKI Panel 2024 (Q2 + Q3)	54.3 % (53.2 – 55.4)	58.1 % (56.8 – 59.5)	50.2 % (48.6 – 51.8)	33.9 % (31.3 – 36.5)	42.4 % (40.3 – 44.6)	60.5 % (59.0 – 62.1)	67.2 % (65.4 – 69.0)	73.3 % (70.2 – 76.3)	57.5 % (54.9 – 60.0)	52.6 % (51.2 – 54.0)	48.6 % (47.0 – 50.2)
	GEDA 2024	53.2 % (50.7 – 55.7)	55.7 % (52.2 – 59.1)	50.8 % (47.2 – 54.4)	32.5 % (26.1 – 39.7)	43.6 % (37.6 – 49.7)	60.0 % (56.0 – 63.7)	67.3 % (63.1 – 71.3)	60.9 % (53.8 – 67.5)	47.0 % (38.4 – 55.7)	55.3 % (51.7 – 58.8)	43.5 % (40.3 – 46.8)
	Missing values (n,% weighted)°	91 (0.7)	57 (0.9)	34 (0.4)	4 (0.2)	5 (0.3)	18 (0.5)	31 (0.8)	33 (3.0)	41 (1.0)	31 (0.4)	16 (0.4)
		10 (0.1)	5 (0.1)	5 (0.1)	0 (0.0)	1 (0.1)	2 (0.1)	6 (0.3)	1 (0.0)	1 (0.0)	3 (0.1)	6 (0.3)
	p-value*	0.44	0.50		0.36					0.21		
	p-value within groups		0.1900	0.76	0.72	0.73	0.78	0.97	0.0006	0.0231	0.1748	0.0069
Obesity BMI ≥ 30 (n = 18,450/3,918)	RKI Panel 2024 (Q2 + Q3)	22.4 % (21.4 – 23.4)	21.6 % (20.4 – 22.9)	23.2 % (21.9 – 24.6)	14.8 % (12.5 – 17.5)	22.7 % (20.8 – 24.8)	25.8 % (24.3 – 27.4)	24.9 % (23.2 – 26.7)	17.4 % (15.0 – 20.1)	30.0 % (27.4 – 32.8)	21.9 % (20.8 – 23.0)	12.9 % (11.8 – 14.0)
	GEDA 2024	19.4 % (17.5 – 21.5)	17.4 % (14.9 – 20.3)	21.5 % (18.5 – 24.7)	8.2 % (5.0 – 13.1)	19.1 % (14.5 – 24.8)	23.2 % (19.9 – 26.8)	24.3 % (20.2 – 28.8)	15.8 % (11.1 – 22.0)	30.3 % (22.2 – 40.0)	18.7 % (16.0 – 21.6)	12.7 % (10.7 – 14.9)
	Missing values (n,% weighted)°	154 (1.1)	98 (1.4)	55 (0.8)	20 (0.9)	16 (0.6)	44 (1.1)	39 (1.0)	35 (3.0)	59 (1.8)	62 (0.8)	33 (0.5)
		98 (2.7)	74 (4.2)	23 (1.1)	12 (3.2)	13 (2.8)	33 (2.1)	17 (1.5)	23 (6.6)	18 (4.8)	48 (2.7)	32 (1.6)
	p-value*	0.0119	0.0126		0.0097					0.24		
	p-value within groups		0.0081	0.31	0.0179	0.22	0.1839	0.78	0.61	0.95	0.0448	0.89
Smoking Yes, daily/Yes, occasionally (n = 18,318/4,014)	RKI Panel 2024°° (Q2 + Q3)	23.7 % (22.7 – 24.6)	20.4 % (19.2 – 21.7)	27.0 % (25.6 – 28.4)	27.1 % (24.6 – 29.7)	30.9 % (28.7 – 33.2)	26.3 % (24.7 – 28.0)	15.3 % (13.8 – 16.9)	3.7 % (2.6 – 5.1)	36.6 % (33.7 – 39.6)	23.4 % (22.3 – 24.5)	13.6 % (12.3 – 14.9)
	GEDA 2024	27.0 % (24.7 – 29.5)	21.9 % (18.9 – 25.2)	32.3 % (28.9 – 36.0)	29.2 % (23.0 – 36.4)	35.5 % (29.8 – 41.7)	30.1 % (26.3 – 34.3)	19.5 % (15.9 – 23.7)	4.5 % (2.4 – 8.4)	38.7 % (30.3 – 47.8)	28.3 % (25.1 – 31.8)	16.0 % (13.5 – 18.8)
	Missing values (n,% weighted)°	286 (1.7)	187 (2.3)	99 (1.1)	18 (0.7)	19 (0.9)	36 (0.8)	106 (2.6)	107 (7.7)	133 (2.6)	89 (1.1)	57 (1.0)
		2 (0.0)	2 (0.1)	0 (0.0)	0 (0.0)	0 (0.0)	1 (0.1)	1 (0.0)	0 (0.0)	0 (0.0)	2 (0.0)	0 (0.0)
	p-value*	0.0088	0.0080		0.0050					0.0132		
	p-value within groups		0.39	0.0046	0.55	0.1488	0.0761	0.0386	0.58	0.65	0.0042	0.0968
Self-rated mental health Excellent/very good (n = 18,042/4,004)	RKI Panel 2024 (Q2 + Q3)	44.9 % (43.9 – 45.9)	40.4 % (39.1 – 41.8)	49.6 % (48.1 – 51.0)	41.8 % (39.0 – 44.6)	45.6 % (43.5 – 47.8)	47.0 % (45.3 – 48.7)	48.3 % (46.4 – 50.2)	32.9 % (29.8 – 36.3)	38.0 % (35.3 – 40.7)	45.8 % (44.4 – 47.2)	54.2 % (52.5 – 55.8)
	GEDA 2024	39.6 % (37.2 – 42.0)	35.4 % (32.3 – 38.7)	43.8 % (40.3 – 47.3)	45.9 % (38.7 – 53.2)	45.3 % (39.5 – 51.2)	40.6 % (36.9 – 44.4)	36.6 % (32.5 – 40.9)	15.2 % (11.7 – 19.4)	29.7 % (21.9 – 38.9)	39.2 % (35.8 – 42.7)	53.6 % (50.3 – 56.9)
	Missing values (n,% weighted)°	180 (1.2)	96 (1.1)	84 (1.2)	57 (2.3)	39 (1.0)	34 (0.7)	21 (0.8)	29 (1.9)	48 (1.5)	86 (1.1)	46 (0.9)
		12 (0.3)	8 (0.3)	4 (0.4)	0 (0.0)	0 (0.0)	3 (0.1)	5 (0.6)	4 (2.2)	6 (0.5)	4 (0.1)	2 (0.1)
	p-value*	< 0.0001	< 0.0001		< 0.0001					0.0003		
	p-value within groups		0.0053	0.0033	0.30	0.91	0.0028	< 0.0001	< 0.0001	0.0868	0.0006	0.78
Depressive symptoms PHQ-8 ≥ 10 (n = 17,856/3,841)	RKI Panel 2024 (Q2 + Q3)	20.7 % (19.8 – 21.6)	23.2 % (22.0 – 24.5)	17.8 % (16.6 – 19.1)	32.2 % (29.6 – 34.9)	22.7 % (20.8 – 24.8)	19.7 % (18.3 – 21.1)	11.1 % (9.8 – 12.5)	17.1 % (14.2 – 20.4)	28.8 % (25.9 – 31.8)	19.7 % (18.6 – 20.8)	14.3 % (13.2 – 15.6)
	GEDA 2024	15.1 % (13.1 – 17.3)	15.6 % (12.8 – 18.7)	14.7 % (11.9 – 18.0)	15.9 % (11.3 – 21.7)	16.4 % (11.9 – 22.3)	16.7 % (13.4 – 20.6)	8.2 % (5.3 – 12.4)	18.2 % (12.2 – 26.3)	18.7 % (12.2 – 27.5)	16.1 % (13.3 – 19.4)	9.2 % (7.2 – 11.7)
	Missing values (n,% weighted)°	366 (2.4)	207 (2.7)	159 (2.0)	39 (1.9)	23 (0.6)	56 (1.3)	135 (4.1)	113 (8.9)	159 (3.1)	135 (1.7)	65 (1.3)
		175 (4.4)	108 (4.3)	67 (4.4)	7 (1.9)	11 (3.0)	33 (2.9)	71 (7.8)	53 (11.1)	52 (4.7)	62 (3.3)	60 (2.9)
	p-value*	< 0.0001	< 0.0001		< 0.0001					0.0004		
	p-value within groups		< 0.0001	0.0768	< 0.0001	0.0443	0.1477	0.1687	0.78	0.0317	0.0428	0.0005
Angstsymptome GAD-2 ≥ 3 (n = 18,039/3,970)	RKI-Panel 2024 (Q2 + Q3)	15.4 % (14.7 – 16.3)	18.4 % (17.3 – 19.5)	12.3 % (11.3 – 13.3)	27.3 % (24.7 – 30.1)	17.8 % (16.2 – 19.6)	13.8 % (12.7 – 15.1)	7.6 % (6.6 – 8.7)	9.8 % (7.7 – 12.4)	17.7 % (15.3 – 20.3)	15.9 % (15.0 – 17.0)	13.7 % (12.5 – 15.0)
	GEDA 2024	13.2 % (11.3 – 15.3)	14.6 % (11.9 – 17.7)	11.8 % (9.3 – 14.8)	16.2 % (11.9 – 21.8)	17.9 % (13.0 – 24.0)	13.4 % (10.5 – 17.1)	5.4 % (3.5 – 8.2)	10.7 % (6.1 – 18.1)	16.8 % (10.6 – 25.6)	13.2 % (10.6 – 16.4)	10.9 % (8.6 – 13.7)
	Missing values (n,% weighted)°	183 (1.3)	103 (1.4)	80 (1.1)	42 (1.7)	26 (0.7)	28 (0.7)	42 (1.3)	45 (4.0)	71 (1.8)	71 (0.8)	39 (0.9)
		46 (1.5)	32 (1.9)	14 (1.0)	2 (1.5)	5 (0.9)	8 (0.4)	11 (0.7)	20 (8.7)	14 (2.0)	16 (0.9)	16 (0.8)
	p-value*	0.0502	0.0619		0.0500					0.1003		
	p-value within groups		0.0260	0.76	0.0007	0.99	0.82	0.1198	0.79	0.83	0.1024	0.0646

°Number and proportion of missing values: The first row represents the RKI Panel 2024, the second row represents GEDA 2024.

°°In the RKI Panel 2024, the smoking status of people with diabetes mellitus was taken from the recruitment study.

*p-value for difference between RKI Panel 2024 (Q2 + Q3) and GEDA 2024 overall (without adjustment) and overall adjusted for gender/age group/education

BMI = body mass index, PHQ-8 = Patient Health Questionnaire-8, GAD-2 = Generalised Anxiety Disorder-2

Analysis based on population status as of 31 December 2023, standardised according to European Standard Population (ESP 2013) within the education groups for stratification by education.

**Table 4:** Estimated method effect on prevalence over time in a comparison of study type 1 with study type 2 (prevalence difference and 95 % confidence intervals)

	Gender identity			Age groups (years)					Education group		
	Total	Female	Male	18 – 29	30 – 44	45 – 64	65 – 79	≥ 80	Low	Medium	High
Self-rated general health (very good/good)	-2.8 (- 3.4 – - 2.1)	- 3.1 (- 4.0 – - 2.2)	- 2.6 (- 3.4 – - 1.7)	- 3.9 (- 5.4 – - 2.4)	- 3.0 (- 4.3 – - 1.7)	- 0.6 (- 1.7 – 0.4)	- 3.1 (- 4.4 – - 1.9)	- 10.9 (- 13.0 – - 8.7)	- 3.0 (- 4.7 – - 1.2)	- 2.2 (- 3.0 – - 1.3)	- 2.1 (- 3.0 – - 1.2)
Chronic conditions (yes)	2.8 (2.1 – 3.5)	2.9 (2.0 – 3.8)	2.9 (1.9 – 3.8)	1.9 (0.2 – 3.7)	0.3 (- 1.2 – 1.8)	2.7 (1.7 – 3.8)	4.2 (3.0 – 5.4)	12.2 (10.2 – 14.1)	2.7 (0.9 – 4.5)	2.7 (1.8 – 3.6)	3.8 (2.8 – 4.8)
Long-term activity limitations (yes, severe or moderate for at least 6 months)	2.5 (0.7 – 4.4)	2.4 (- 0.1 – 5.0)	2.8 (0.1 – 5.5)	1.2 (- 3.7 – 6.1)	4.0 (0.3 – 7.8)	1.9 (- 1.3 – 5.0)	2.8 (- 0.7 – 6.3)	4.9 (- 0.5 – 10.2)	- 4.8 (- 10.4 – 0.8)	3.7 (1.2 – 6.1)	2.4 (0.1 – 4.8)
Impairments in physical function (yes, great difficulty or inability to walk or climb stairs)	- 1.2 (- 1.8 – - 0.6)	- 1.5 (- 2.4 – - 0.5)	- 0.9 (- 1.7 – - 0.0)	0.7 (- 0.0 – 1.5)	- 0.4 (- 1.4 – 0.6)	- 3.3 (- 4.5 – - 2.0)	- 2.9 (- 4.7 – - 1.1)	4.5 (0.5 – 8.5)	- 2.7 (- 4.5 – - 1.0)	- 1.2 (- 2.0 – - 0.3)	- 0.2 (- 0.8 – 0.5)
Diabetes (yes, ever medically diagnosed)	0.2 (- 1.0 – 1.4)	- 0.6 (- 1.8 – 0.6)	0.6 (- 1.2 – 2.4)	0.3 (- 1.6 – 2.2)	0.0 (- 1.5 – 1.6)	- 2.1 (- 3.9 – - 0.2)	0.3 (- 2.2 – 2.8)	- 0.7 (- 4.9 – 3.6)	- 3.2 (- 5.7 – - 0.6)	- 0.2 (- 1.6 – 1.1)	1.2 (0.1 – 2.3)
Obesity (BMI ≥ 30 kg/m^2^, calculated from self-report)	1.9 (1.3 – 2.5)	2.1 (1.3 – 2.9)	1.7 (0.8 – 2.5)	2.4 (1.0 – 3.8)	3.1 (1.8 – 4.4)	1.0 (0.0 – 2.0)	2.2 (1.1 – 3.3)	0.2 (- 1.4 – 1.9)	1.6 (- 0.1 – 3.3)	2.2 (1.4 – 3.0)	1.0 (0.3 – 1.8)
Smoking (yes, daily or yes, occasionally)	- 4.6 (- 5.3 – - 3.9)	- 3.5 (- 4.4 – - 2.6)	- 5.7 (- 6.7 – - 4.7)	- 5.2 (- 7.0 – - 3.4)	- 4.1 (- 5.6 – - 2.7)	- 4.7 (- 5.8 – - 3.7)	- 6.5 (- 7.8 – - 5.3)	- 1.3 (- 2.3 – - 0.3)	- 4.1 (- 6.0 – - 2.1)	- 5.4 (- 6.3 – - 4.5)	- 2.9 (- 3.8 – - 2.0)
Self- rated mental health (Excellent/very good)*	- 4.6 (- 6.3 – - 3.0)	- 5.9 (- 8.1 – - 3.6)	- 3.5 (- 5.9 – - 1.1)	- 5.2 (- 9.9 – - 0.5)	- 5.9 (- 9.7 – - 2.1)	- 4.7 (- 7.3 – - 2.1)	- 3.2 (- 6.0 – - 0.3)	- 1.6 (- 4.7 – 1.5)	- 0.0 (- 5.0 – 5.0)	- 3.6 (- 5.9 – - 1.4)	- 8.7 (- 11.0 – - 6.4)
Depressive symptoms (PHQ-8 ≥ 10)	4.2 (2.6 – 5.9)	6.0 (3.7 – 8.3)	2.2 (- 0.1 – 4.5)	11.2 (6.4 – 16.0)	4.4 (0.7 – 8.0)	2.6 (- 0.0 – 5.2)	2.6 (1.2 – 4.0)	- 0.1 (- 5.7 – 5.6)	3.7 (- 1.5 – 8.9)	3.6 (1.5 – 5.7)	6.6 (5.5 – 7.7)
Anxiety symptoms (GAD-2 ≥ 3)	2.3 (0.5 – 4.2)	3.2 (0.5 – 5.9)	1.2 (- 1.3 – 3.6)	9.2 (4.1 – 14.3)	1.0 (- 3.6 – 5.6)	0.2 (- 2.8 – 3.1)	1.3 (- 0.7 – 3.4)	1.8 (- 2.6 – 6.2)	- 1.1 (- 7.2 – 5.1)	3.0 (0.6 – 5.4)	4.9 (2.6 – 7.1)
Social support (strong support)	- 11.2 (- 11.9 – - 10.5)	- 12.3 (- 13.3 – - 11.3)	- 10.0 (- 11.0 – - 9.0)	- 13.1 (- 14.8 – - 11.4)	- 14.4 (- 15.9 – - 12.8)	- 11.2 (- 12.3 – - 10.0)	- 6.8 (- 8.3 – - 5.3)	- 2.6 (- 5.1 – - 0.1)	- 8.6 (- 10.5 – - 6.7)	- 12.3 (- 13.3 – - 11.2)	- 14.1 (- 15.3 – - 13.0)

Data basis: All surveys from GSTel03 to RKI Panel 2024 that contain the respective indicator, including the GEDA 2024 methodological study and the RKI Panel 2024 recruitment study.

*The data from the annual RKI Panel 2024 survey were not used in the trend analysis for the indicator ‘Self-rated mental health’ due to identified questionnaire effects in the parallel comparison.

Analysis with age and gender standardisation according to the European Standard Population, for stratification by education also within the education groups.

Comparison of study type 1 (registry sample, self-administered questionnaire) to study type 2 (telephone sample with telephone interview)

Method effect = model-based difference in prevalence between study type 1 and study type 2; method effect > 0 indicates that the prevalence is higher in study type 1.

BMI = Body Mass Index, PHQ-8 = Patient Health Questionnaire-8, GAD-2 = Generalised Anxiety Disorder-2

**Annex Table 1:** Overview of the methodology of the surveys used

Survey	Sampling		Survey mode	Field time	Number of cases^a^	Response rate^b^	Weighting			
	Residents’ registration office (EMA) sample	Telephone sample: Landline (F)/Dual-Frame (DF)	Telephone interview 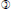 /Mixed mode written (web-based 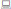 /paper 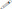 )		Number of participants aged 18 and older		Population structure as of 31 December	Education/proportion of single households according to microcensus	Adjustment weighting based on …	Education classified according to …
GSTel03 (2003) [1,2]		F	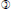	9/2002 to 3/2003	8,318	Based on RR2: 53.8 %	2002	2003	Gender, age, region, education	ISCED 1997 [18]
GSTel06 (2006) [3]		F	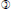	10/2005 to 3/2006	5,542	Based on RR2: 37.4 %	2006	2007	Gender, age, region, education	ISCED 1997 [18]
GEDA 2009 [4–6]		F	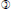	7/2008 to 5/2009	21,262	RR2: 25.7 %	2007	2007	Gender, age, federal state, education	ISCED 1997 [18]
GEDA 2010 [4, 6, 7]		F	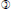	9/2009 to 7/2010	22,050	RR2: 25.3 %	2008	2008	Gender, age, federal state, education	ISCED 1997 [18]
GEDA 2012 [4, 6, 8]		F	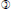	3/2012 to 2/2013	19,294	RR2: 20.5 %	2011	2010	Gender, age, federal state, education	ISCED 1997 [18]
GEDA 2014/2015-EHIS [4, 9, 10]	EMA		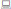 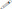	11/2014 to 7/2015	24,016	RR1: 27.6 %	2014	2013	Gender, age, federal state, education, settlement structure (district type)	ISCED 2011 [19]
GEDA 2019/2020-EHIS [4, 11]		DF	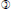	4/2019 to 9/2020	22,708	RR3: 21.6 %	2019	2017	Gender, age, federal state, education, settlement structure (district type)	ISCED 2011 [19]
GEDA 2022 [4, 12]		DF	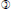	2/2022 to 1/2023	33,149^c^	RR3: 16.1 % – 19.6 %	2020	2018	Gender, age, federal state, education, settlement structure (district type) [Weighting per survey date and module, if applicable]	ISCED 2011 [19]
GEDA 2023 [4, 12]		DF	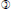	1/2023 to 2/2024	30,002^c^	RR3: 17.4 % – 19.8 %	2020	2018	Gender, age, federal state, education, settlement structure (district type) [Weighting per survey date and module, if applicable]	ISCED 2011 [19]
RKI Panel 2024 Recruitment Study [13–15]	EMA		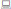 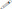	1/2024 to 5/2024	61,460	RR2: 37.6 %	2023	2021	Gender, age, federal state, education, settlement structure (BIK municipality type [22]), proportion of single households [16]	CASMIN [20, 21]
GEDA 2024 (methodological study, parallel survey) [4]		DF	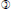	6/2024 to 9/2024	4,016	RR3: 19.6 % – 20.0 %	2023	2021	Gender, age, federal state, education, settlement structure (district type)	ISCED 2011 [19]
RKI Panel 2024 [13, 14, 17]	EMA		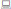 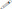	6/2024 to 1/2025	FB A: 27,199 FB B: 27,147 FB C: 27,102 FB D: 27,306	RR2: 77.7 % – 78.1 % RR2cum: 22.3 % – 22.4 %	2023	2021	Gender, age, federal state, education, settlement structure (BIK municipality type [22]), proportion of single households; additional compensation for dropout in the sub-waves based on data from the recruitment study [16]	CASMIN [20, 21]

FB = questionnaire. The term ‘region’ refers to the grouping of federal states; this was done differently in the individual surveys.

^a^The case numbers given here refer to the age group 18 and above.

^b^Response rates RR1, RR2, RR3 and RR2cum according to AAPOR [23]. In the RKI Panel, RR2 stands for the re-participation rate of registered panellists and RR2cum for the cumulative response rate based on those invited to participate in the recruitment study.

^c^GEDA 2022 and GEDA 2023: total number of participants across all survey sub-waves and all modules

**Annex Table 2:** Expected direction of method effects (differences in prevalence estimates) depending on study design

Indicator	Social desirability	Fact question (F), assessment (E)	Likert scale	Number of items	Expected trend	Explanation of the expected direction of prevalence differences depending on the study design:
Good/very good self-rated general health	Low	E	Yes, 5-level	1	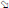	The proportion of people with good/very good health is slightly lower in the RKI Panel 2024 than in GEDA due to the Likert scale response format; this may be more pronounced in the age group ≥ 80^*^.
Chronic conditions	Low	F	No, binary (yes/no)	1	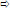 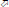	The proportion of chronically ill persons is higher in the RKI Panel 2024 than in GEDA; possibly more pronounced in the age group ≥ 80^*^.
Long-term activity limitations	Minimal	E + F	Yes, 3 levels	2	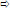 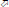	The proportion of people who have been moderately or severely restricted for at least 6 months does not differ; possibly higher prevalence in the age group ≥ 80^*^.
Impairments in physical function	Low	E	Yes, 4 levels	2	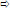 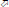	The proportion of people with great difficulty or inability to walk or climb stairs does not differ; possibly higher prevalence in the age group ≥ 80^*^.
Diabetes mellitus	Low	F	No, binary (yes/no)	1	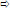 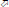	The proportion of people with medically diagnosed diabetes mellitus does not differ; possibly higher prevalence in the age group ≥ 80^*^.
Obesity	High	F	No, binary (yes/no)	2	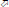	The proportion of people with obesity (self-reported BMI ≥ 30 kg/m^2^) is higher in the RKI Panel 2024, as the effect of social desirability is lower in written surveys. In addition, the panel offers the option of measuring height and weight, which tends to result in more accurate information.
Smoking	Medium	F	No, categorical	1	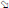	The proportion of people who smoke daily or occasionally is lower in the RKI Panel, as more health-conscious individuals may participate in a panel on health topics.
Excellent/very good self-rated mental health	Average	E	Yes, 5-point scale	1	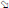	The proportion of people with excellent/very good mental health is slightly lower in the RKI Panel 2024 than in GEDA due to the Likert scale response format or selection effects^**^.
Depressive symptoms	High	E	Yes, 4-level	8	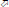	The proportion of people with depressive symptoms (PHQ-8 ≥ 10) is higher in the RKI Panel 2024 than in GEDA, mainly due to the multiple Likert scale response format and because the effect of social desirability is lower in written surveys, possibly with additional selection effects^**^.
Anxiety symptoms	High	E	Yes, 4-level	2	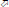	The proportion of people with anxiety symptoms (GAD-2 ≥ 3) is higher in the RKI Panel 2024 than in GEDA, mainly due to the multiple Likert scale response format and because the effect of social desirability is lower in written surveys, possibly with additional selection effects^**^.
Social support	Medium	E	Yes, 5-point scale	3	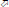	The proportion of people with strong social support (OSSS-3 ≥ 11) is lower in the RKI Panel 2024 than in GEDA, mainly due to the multiple Likert scale response format and because the effect of social desirability is lower in written surveys.

Notes: GEDA = German Health Update, BMI = Body Mass Index, PHQ-8 = Patient Health Questionnaire-8, GAD-2 = Generalised Anxiety Disorder-2, OSSS-3 = Oslo 3-item Social Support Scale

^*^Sampling effects: more people with poorer health in the RKI Panel among older people

^**^Sampling effects: It is possible that more people with mental health issues participate in a panel on health topics, especially in a questionnaire focused primarily on mental health.
